# Recent trends in (bio)analytical chemistry

**DOI:** 10.1007/s00216-021-03524-z

**Published:** 2021-07-28

**Authors:** Günter Gauglitz, Antje J. Baeumner

**Affiliations:** 1 Institute for Theoretical and Physical Chemistry, Eberhard-Karls-University, Tübingen, Germany; 2 Institute for Analytical Chemistry, Bio- and Chemosensors, University Regensburg, Universitätsstraße 31, 93053 Regensburg, Germany

The Analytica conference has been established as part of the biennial Analytica Munich, the world’s leading trade fair for laboratory technology and methodology in the analytical sciences. The Analytica conference is an interdisciplinary and future-oriented event, and combines a practice-oriented program with lectures about fundamental and innovative developments in instrumentation, detection, separation, and validation methods. The conference’s slogan is “Science meets Industry.” Consequently, its purpose is to bring together researchers, users, and manufacturers to promote the transfer and exchange of knowledge.

The 2020 Analytica conference was originally scheduled for March/April. Three scientific societies, the German Society for Biochemistry and Molecular Biology (GBM), the German Society for Clinical Chemistry and Laboratory Medicine (DGKL), and the German Chemical Society (GDCh), had organized approx. 180 lectures in more than 40 sessions on topics including data mining, machine learning, in-line analytics, smart lab, imaging, artificial intelligence, e-health, and analytics 4.0 [1]. However, the outbreak of COVID-19 forced the event, like so many others in 2020, to be postponed until October when Analytica 2020 was launched as *Analytica virtual—*“the biggest digital event in 2020 for laboratory technology analysis and biotechnology” with more than 300 interactive virtual exhibition stands and 200 webinars. The event became a success as half of the original speakers agreed to screen-cast their talks. These could be streamed twice a day at different times enabling the participation of a broad global audience. Albeit a success, we editors of ABC learned from the scientific community that authors and audience experienced a lack of information transfer due to busy schedules at the office, missing networking opportunities in person and all of the shortcomings digital events have in comparison to in-person meetings. Accordingly, ABC decided to provide an additional platform to the speakers of this conference and invited a selection of renowned authors in the field for a topical collection “Recent Trends in (Bio)analytical Chemistry” whose contributions have been successfully peer-reviewed.

These papers cover the wide range of analytical science published in ABC, such as modern development in spectroscopy, separation science, sensor technology, and omics. For example, Raman spectroscopy is used to compare functional and discrete data analysis regimes. Mass spectrometry is discussed fundamentally by observing charged droplets from electrospray ionization (ESI) plumes, in combination with different chromatographic and sampling modes for high-resolution mass spectrometric screening of organic microcontaminants in water, as well as through applied studies with intact plasma quantification of the large therapeutic lipopeptide bulevirtide. Other applied investigations include method development, validation, and reference population-derived thresholds of carbon isotope ratios of endogenous steroids found in human serum, and recent trends in drugs of abuse metabolism study-based analytical screening procedures. Sensors and POCT applications are the basis for integrating high-performing electrochemical transducers in lateral flow assay, or the detection of plant virus particles with a capacitive field-effect sensor, as well as the evaluation of the use of dried blood spot and microsampling specimens to avoid post-sampling formation of phosphatidylethanol formed in cell membranes following alcohol consumption. The discussion of an automated, flow-based chemiluminescence microarray immunoassay for the rapid multiplex detection of IgG antibodies to SARS CoV 2 in human serum and plasma (CoVRapid CL MIA) is more than timely these days, as are the discussions of trends in the POCT-field such as those from bedside to bench—practical considerations enabling pre-analytical pitfalls to be uncovered. The important field of “omics” is represented through a critical review that assesses the suitability of sample quality for high-resolution metabolomics and lipidomics analyses of body fluids, and trends in peptide-array based interactomics discussing the understanding of cellular signaling.

We are happy that we succeeded in publishing this topical collection in less than one year after the event, and hope that you, the readers, will experience the conference’s slogan “Science meets Industry”—reflected in these papers. Thus, we hope you will enjoy this ABC issue. Stay tuned to see if we will repeat such a topical collection in parallel to future Analytica conferences—and we hope to see you in person at the next event, maybe even at an ABC-sponsored symposium.
